# Quality of Care during Neonatal Resuscitation in Kakamega County General Hospital, Kenya: A Direct Observation Study

**DOI:** 10.1155/2017/2152487

**Published:** 2017-10-29

**Authors:** Duncan N. Shikuku, Benson Milimo, Elizabeth Ayebare, Peter Gisore, Gorrette Nalwadda

**Affiliations:** ^1^Department of Nursing, School of Health Sciences, Makerere University, Kampala, Uganda; ^2^Department of Midwifery and Gender, School of Nursing, Moi University, Eldoret, Kenya; ^3^Department of Child Health and Pediatrics, School of Medicine, Moi University, Eldoret, Kenya

## Abstract

**Background:**

Birth asphyxia is the leading cause of neonatal mortality in Kenya. Quality care during neonatal resuscitation (NR) can contribute to a reduction in neonatal mortality related to birth asphyxia by 30 percent. This study assessed the quality of care (QoC) during NR for newborns with birth asphyxia.

**Methods:**

Direct observations of 138 newborn resuscitations were done in labor ward and maternity theatre. Twenty-eight healthcare providers were observed 3–5 times using a structured checklist. Descriptive and inferential statistics were calculated and quality of care scores computed. Ordered logistic regression model identified HCPs characteristics associated with the QoC scores during NR.

**Results:**

Overall QoC scores were good for airway clearance (83%). Suctioning in meconium presence (40%) was poorly performed. Years of experience working in maternity were associated with good drying/stimulation (*β* = 1.86, *P* = 0.003, CI = 0.626–3.093) and airway maintenance (*β* = 1.887, *P* = 0.009, CI = 0.469–3.305); nurses were poor compared to doctors during initial bag and mask ventilation (*β* = −2.338, *P* = 0.05, CI = −4.732–0.056).

**Conclusion:**

Key steps in NR are poorly performed during drying and warmth, airway maintenance in meconium presence, and ventilation. Mentorship with periodic refresher training can improve the care provided during NR.

## 1. Background

Globally, approximately 4 million deaths occur in neonates with 99 percent of them occurring in low and middle income countries [1, 2]. Global neonatal mortality rate stands at 21 per 1000 live births, with African region at 32 per 1000 live births [3] where Sub-Saharan Africa alone approximates to about 77% of the neonatal deaths [4]. Birth asphyxia has for a long time been estimated to account for approximately 25 percent of neonatal mortality worldwide [5]. This is despite the morbidity and mortality from birth asphyxia being preventable and treatable [6].

The majority of the neonatal deaths (75 percent) occur in the first week of life, with approximately half of these occurring within the first 24 hours after birth [2, 5, 7]. Significantly, the risk of death increases by 16 percent for every 30 seconds delay in initiating ventilation up to six minutes and every six percent for every minute of delay of applied bag and mask ventilation [5]. Evidence has shown that training of healthcare providers in Helping Babies Breathe intervention can contribute to a 47% reduction in early neonatal mortality [8]. This points to the need for appropriate care of these babies especially during the critical first hours of life. Therefore, efforts to improve child health have to focus on reducing neonatal deaths, in particular early deaths within the first minutes.

Effective NR could prevent neonatal deaths by 30 percent as well as improve the outcomes of newborns delivered with birth asphyxia [9]. This has been demonstrated in low resource settings in Sub-Saharan Africa and developing world [10].

**Figure 1:**
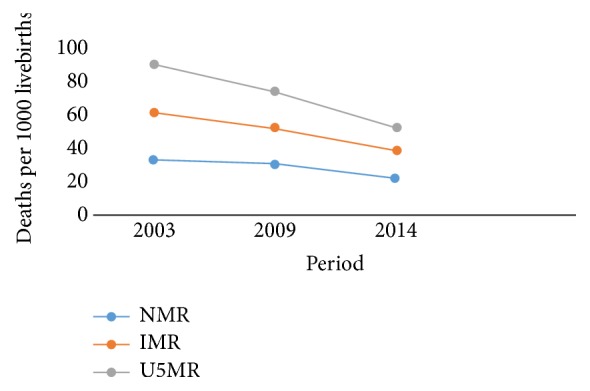
Early childhood mortality trends in Kenya.* Source*.* Kenya Demographic and Health Survey 2014*. NMR: Neonatal Mortality Rate, IMR: Infant Mortality Rate, U5MR: Under 5 Mortality Rate.

In Kenya, neonatal mortality rate is still high, currently at 22 per 1000 live births [11], a significant decline from 31 per 1000 live births in 2008-09 [12]. Importantly, there was an increase in skilled deliveries from 44 to 62 percent during the same period. However, this national neonatal mortality rate is still above the sustainable development goal target of 3.2 that aims to end preventable deaths of newborns and children under 5 years of age, with all countries aiming to reduce neonatal mortality to at least as low as 12 per 1,000 live births and under – 5 mortality to at least as low as 25 per 1,000 live births by 2030 [13]. The slow reduction explains the 60 percent infant deaths occurring during the first year of life from neonatal causes. This is in contrast to significant reductions noted in the infant and under five mortality rates [11] (Figure 1).

Birth asphyxia remains the leading cause of neonatal mortality in Kenya at 29 percent [14]. Skills of health professionals regarding NR are very crucial to ensure good immediate neonatal outcome [6, 15]. The MOH (Kenya) recognizes the importance of NR services as part of the Kenya Basic Paediatric Protocols and basic emergency obstetric and neonatal care (BEmONC) from level 2 health facilities [14, 16].

Measures of quality focus on the structure, process, and outcomes [17]. However, there is limited data on the attention paid to evaluating the quality and practices of care in public hospital settings. Previous research studies indicate that despite availability of neonatal resuscitation protocols for use in delivery rooms, approximately 30 percent of the steps are either wrongly performed or not performed [18–20]. Elsewhere, NR is often not initiated or the methods used are inadequate or wrong making it ineffective [21]. The MOH (Kenya) has made attempts to provide in-service emergency care NR training to HCPs currently as a strategy to improve the outcomes of newborns with birth asphyxia [22].

Despite HCPs' preservice and in-service training, guidelines, and job aids with adequate equipment, practices of HCPs with regard to NR are still reported to be poor [23–25]. Ineffective or wrong resuscitation practices are linked to the persistently high neonatal deaths from birth asphyxia in the first 1–24 hours [22]. This early neonatal mortality and morbidity is a key indicator of the serious lack of quality neonatal services. Quality NR at birth could contribute to reduction in neonatal mortality related to birth asphyxia [10]. However, evidence to this effect is daunting in Kenya. The study aims were to assess the quality of care provided during the NRs in order to contribute towards evidence-based efforts to reduce neonatal deaths attributable to birth asphyxia.

## 2. Materials and Methods 

### 2.1. Study Design

This was a cross-sectional study employing direct observations of NR in labor ward and maternity theatre. This method is nonintrusive, where the HCPs do what they normally do (resuscitation) without being interrupted or disturbed by the observer [26, 27]. This allowed the HCPs to be observed in their natural fashion without interfering in their process of care provision.

### 2.2. Study Setting

The study was conducted between April and June 2016 at Kakamega County General Hospital's (in Kakamega County, Western Province) labor ward and maternity theatre. However, the newborn unit was excluded as newborns are only referred here after stabilizing from the initial resuscitation done in labor ward or maternity theatre immediately after delivery. This is the main government referral facility in the Western Kenya region also serving as a training centre for trainee nurses and other healthcare professionals. Therefore, it was the ideal location to assess the quality of care during NR as it is a place where there is a high demand for NR and therefore need for updated and evidence-based practices among health care workers.

### 2.3. Study Participants

Twenty-eight HCPs who conducted NRs who met the inclusion criteria and voluntarily consented to participate in the study were recruited. The HCPs must have been working in the labor ward and/or the maternity theatre and providing direct NR services. Trainee nursing and medical students were excluded (unlicensed to practice).

Based on the WHO and the American Academy of Paediatrics, newborns who met the inclusion criteria were recruited [28, 29]. This included failure to initiate spontaneous respirations at birth/within 1 minute of delivery (no breathing at 1 minute of age) and/or gasping breathing at 30 seconds after birth and/or baby is floppy and/or bluish or has central cyanosis (blue tongue). Stillbirths and those with congenital abnormalities incompatible with life were excluded.

### 2.4. Sampling and Sample Size Calculation

All HCPs who were involved in resuscitating a newborn and consented to participate in the study were included. Consecutive sampling was used to select all the newborns that required NR immediately after birth and met the inclusion criteria until the required sample size was achieved.

It is estimated that about 1 in 10 babies needs help to breathe immediately after birth and, therefore, a quick assessment immediately after birth remains the best way to know if a baby needs help to breathe [30]. The Kish Leslie formula (**n** = **Z**^2^**pq**/**e**^2^) for cross-sectional studies was used to calculate the sample size of the NRs to be observed [31]. The** Z** (variate from normal distribution that represents the level of confidence) was 1.96;** p** (estimated proportion of attribute present in a population) was set at 10% as the number of newborns who require resuscitation to breathe at birth [30]; and** q** = 1 − *p*. The desired level of precision (**e**) was set at 95% (minimum acceptable errors at 5%) giving a total of 138 as the desired newborn resuscitations to be observed. Each HCP was observed for three to five times providing NR care. Evidence shows that, with the continued presence of the observer over time, the provider tends to forget because of human nature and sees him/her as a “fly on the wall” thus returning back to their routine care, thus the need for repeated observations [32].

### 2.5. Study Procedures

All NRs were performed at a central common resuscitaire serving both the labor ward and theatre. Four research assistants (RAs) were recruited and observed the NRs for both day and night shifts. The RAs were recruited from among the hospital nurses from the antenatal ward to minimize the Hawthorne effect. This was on the assumption that the HCPs were less likely to change their practices when being observed by another HCP in the same unit as opposed to an observer from outside the hospital. The RAs were nurses with experience and formal NR training. Thus, they were not given any formal NR training by the principal researcher. However, they were given a two-day overview on Helping Babies Breath and the national NR guidelines principles that were utilized in observing the resuscitations against a predetermined checklist. Two practical observations were done with the principal researcher to ensure that all the RAs used the checklist in a similar manner.

#### 2.5.1. Preparation Phase

HCPs were sensitized on the study and its significance with emphasis on entirely voluntary participation. However, due to different working shifts, most of the HCPs had to be approached on an individual basis by the RAs when they were on duty. Upon agreeing to participate in the study, the HCP was assigned with a participant study code (to protect the HCP's identity) and requested to complete a written consent form. The HCP was also requested to complete the structured observation checklist on the biodata form including information on training and qualifications, working experience, and refresher training attended by the research assistant. Importantly, HCPs were sensitized once of the researcher's intent to observe the actual resuscitation during the consent process without subsequent reminders during the actual procedure.

Research assistants were briefed and advised to watch out for any inappropriate/harmful practices during NR. These included holding the neonate upside down, shaking the neonate vigorously, hard patting/slapping of neonate on back, flicking foot of the neonate, vigorously wiping neonate, and squeezing chest of neonate.

#### 2.5.2. Data Collection

An RA was present every time a delivery was being conducted either in theatre or delivery room since NR is a rapid process that required the availability of the study team member at any time. In case of two resuscitations occurring at the same time, the observer proceeded with the delivery that began first. However, this situation only occurred twice. The observer strategically positioned herself near the resuscitaire in order to have a clear view of the resuscitation process from the start to end.

Initial data was collected about the availability, functionality, and accessibility of the essential NR equipment at the resuscitaire. Once a newborn was delivered, the observer included the resuscitation only if two criteria were fulfilled. First, the HCP receiving the newborn had consented to participate in the study and the newborn delivered required resuscitation (met the eligibility criteria). The RA, utilizing a predetermined checklist, observed the actual NR as conducted by the HCP on the ward to assess the skills of the HCP under observation. Importantly, national and international guidelines on NR recommend that a HCP should call for help when urgent need to save the life of newborn arises [16, 30]. Being a direct observation study, the researchers tried their best not interfere in the NR process as it is nonintrusive [26, 27] and focused on the HCPs NR techniques as routinely done.

#### 2.5.3. Data Collection Instrument

A structured direct observation checklist was used. It was sectioned to include the structural characteristics, health facility and the HCP characteristics; the processes, NR process with items based on the principle areas of the NR process as per the national guidelines, preparation for resuscitation, drying/stimulation, airway clearance and maintenance, and bag and mask ventilation (and advanced care/support ventilation) adapted from the Ministry of Health (Kenya), Basic Pediatrics Protocols for ages up to 5 years, Revised, July 2013 Edition [16] for NR.

### 2.6. Data Quality Control and Management

Data collection tools were pretested at the labor ward of old Mulago National & Referral Hospital, Uganda, to maintain their reliability and validity. Random supervisory visits by principal researcher were conducted to ensure that observations were being carried out and checklists were being filled in on site. Regarding areas of uncertainty in the checklist and, in cases where the principal researcher noted any errors in terms of data collection, the error was brought to the attention of the RAs and corrected immediately. At the end of the shifts, forms and checklists were checked by the principal researcher/RA for completeness and errors before leaving the study area/site. The data collected was kept strictly confidential.

### 2.7. Variables and Measurements

The primary outcome was quality of care during NR. It was measured as a continuous variable constructed as a composite variable from the total of 13 step items based on the 4 principle areas of NR process. They were drying/stimulation [three items], checking airway [three items], initial bag and mask ventilation [two items], and advanced bag and mask ventilation [five items] (Table 3). Independent variables expected to influence QoC during NR were summarized as structural/input factors and were cadre, training/qualifications and experience of HCP, and support staff supervision and NR training.

### 2.8. Data Analysis

Raw data was entered in Microsoft Office Excel 2013 software. Data was cleaned, edited to identify any missing values or any other inconsistencies, and exported to STATA version 13 for analysis. The unit of analysis was the resuscitated newborn. All the data from HCPs who had 3–5 observations was analysed. This was a representative and appropriate comparison that eliminated the early initial fears of the HCP of being observed during the practice [32].

A binary score of yes (performed) and no (not performed) was used for all the 13 process variables based on the four principle areas of NR. The variables in each of the four principle areas (indicators of quality) of assessing quality ultimately defined quality of care. Responses from the nominal scale were scored as 1 (for yes) or 0 (for no). Higher scores of responses reflected higher quality for the nominal scale. For process indicators, descriptive statistics were summarized using the mean and standard deviation.

QoC was assessed in detail by analysing performance at each of the four principle steps of resuscitation. QoC was classified as good (if all the recommended steps performed), fair (half the recommended steps performed), and poor (majority of the recommended steps missed).

For each of the four principle steps, we summed the scores of a given HCP for the different steps under that principle step. A new variable was generated to represent a total score of a HCP who attended to a specific neonate for all the specific steps under the principle steps that the neonate received. The new generated variable was used as a measure of quality of care received by the neonate. The score generated was categorized as good, fair, or poor quality. Because of variations in the steps under each of the four principle areas, a specific scoring system for each principle area was adopted. For drying/stimulation and airway clearance (0–3), quality of care was categorized as good if all the three steps, fair if two steps, and poor if one or none of the steps under principle area was performed. For initial bag and mask ventilation (0–2), quality of care was either good if all two steps or poor if one or none of the steps was performed. For advanced/supportive bag and mask ventilation (0–5), quality of care was either good if four or all five steps, fair if three, or poor if only two or less steps were performed. It is important to note that neonates who did not respond to care in the initial steps during resuscitation proceeded to the subsequent levels of resuscitation. Therefore, the quality of care was computed for each of the four principle steps as the number of neonates kept reducing from one level of care to the next.

Descriptive statistics of HCPs' characteristics using frequencies and percentages were computed. Since the dependent variable (quality of care) was ordered, the ordered logistic regression model was used to compare the QoC scores with the HCPs characteristics. This was carried out for each of the four specific principle areas of resuscitation applied. Chest compressions assessment was not analysed in this study as the decision to initiate chest compressions depends on variables not easily determined by the observer and very few resuscitations require chest compressions [33]. Beta coefficients from the ordered logistic models for all independent variables were shown. Positive values of a variable category indicated higher perceived quality relative to the referent category level. Negative values indicated a decreasing perceived quality compared to the reference category. With a 95% confidence interval, *P* values ≤ 0.05 were considered statistically significant.

### 2.9. Ethical Statement

The study protocols were approved by the Makerere University, School of Health Sciences Institutional Review Board (IRB) [SHSREC REF: 2015 – 028], Moi University/Moi Teaching and Referral Hospital (MTRH) Institutional Research and Ethics Committee (IREC) [FAN: IREC 1608], and the Kakamega County General Hospital Ethics and Research Committee [ERC no. 9/04/2016].

Informed written consent was obtained from the HCPs prior to enrolment to participate in the study. There was apprehension on the side of HCPs on repercussions in case of identification of incorrect practices or poor skills. However, they were assured that the records would not bear any identification apart from the study code. In addition, there was strict observation of confidentiality at all levels of the study. To avoid change in practices and alteration of results, HCPs noted to have poor skills were given confidential individual feedback at the end of the 3–5 rounds of observations to protect the interests of the newborns.

## 3. Results

### 3.1. Structure/Inputs

#### 3.1.1. Health Care Providers Background Characteristics

The mean age of HCPs was 32.8 years (SD ± 7.0) with a range of 24 years to 50 years. Majority of HCPs (89.3%) were aged 25 years and above. Nurses/midwives were the majority cadre (71.4%) providing newborn resuscitation. Over half of HCPs (53.6%) providing newborn resuscitation care were registered diploma holders. Two-thirds of nurses/midwives (65%) were registered diploma holders and one-third (35%) were bachelor degree (graduate) nurses. Most of the HCPs (89.3%) had worked in the maternity ward providing NR care for more than a year. Eighteen HCPs (64.3%) reported ever attending a formal NR training. These training courses included Helping Babies Breathe (*n* = 18), European Pediatrics Life Support (*n* = 9), and Pediatric Advanced Life Support (*n* = 11). Most HCPs (66.7%) had such training over 12 months prior to this study as illustrated in Table 1.

Importantly, all the HCPs who participated in the study had at least undergone either a formal NR training or received the initial routine orientation offered to new staffs joining the maternity unit on a number of emergency obstetrics and neonatal care skills, for example, basic neonatal resuscitation, manual removal of the placenta, and maternal resuscitation at the hospital.

#### 3.1.2. Distribution of Newborn Babies during NR

Nurses provided NR for the majority of the newborn babies (72.5%). Diploma holder HCPs cared for over half of the newborn babies (52.9%). Healthcare providers who had undergone a NR training cared for the majority of the newborn babies (63.8%); however the training mostly occurred over a year ago prior to this study (65.9%). Importantly, HCPs who had worked for less than a year in the unit cared for the least (10.9%) whereas those with over 1-year experience in maternity cared for the remainder (89.1%) (Table 1).

#### 3.1.3. Health Facility Characteristics

Helping Babies Breathe (HBB) NR action plans and guidelines were displayed at the resuscitation area. No immediate newborn care and warm chain charts were observed in the unit.

Most of the NR equipment, two resuscitaires equipped with electric warmers; oxygen source (two oxygen cylinders, oxygen flow meters, and oxygen tubing); suction devices (electric suction machine and coloured suction bulbs); ambu bags; preterm and term face masks; and a wall clock were available, functional, and accessible at the resuscitation station. However, only one clean dry towel was present in each delivery pack.

#### 3.1.4. Infection Prevention Practices

Almost all resuscitation cases (*n* = 135, 97.8%) had equipment used (suction devices and face masks) well processed with the HCPs adhering to recommended infection prevention practices. These were cleaning of ventilating face mask and the suction device, decontamination in 0.5% sodium hypochlorite solution for 10 minutes (although occasionally some stayed longer than the 10 minutes), washing with soap and water, and rinsing well and airing to dry until next use. However, no high level disinfection for these equipment was done as it is only performed in theatre using cidex solution and rinsed well with sterile water at the hospital.

### 3.2. Processes of Care

**Figure 2:**
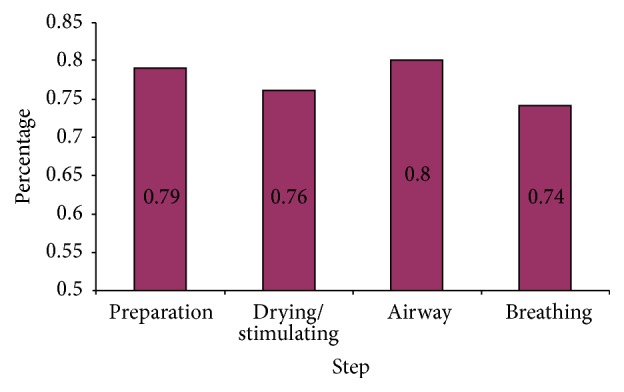
Mean scores for the main steps in neonatal resuscitation at KCGH.

Overall mean scores indicated that airway clearance for baby with no breathing after stimulation at birth was the most commonly performed principle NR step (mean score 0.80, SD ± 0.33) while bag and mask ventilation was the least (mean 0.74, SD ± 0.38) performed (Figure 2).

Nurses were the commonly identified helpers before the resuscitation with over half (*n* = 39, 52.7%) while the anaesthetists (*n* = 19, 25.7%) and the medical officers (*n* = 16, 21.6%) formed the other half.

A few inappropriate stimulation practices observed during the resuscitations included vigorously rubbing the baby's back and chest (*n* = 11, 8%), flicking the baby's feet (*n* = 3, 2.2%), and patting the baby's back (*n* = 1, 0.7%). Inappropriate head positioning was observed in 21 (17%) cases and 11 (7.2%) of the babies were turned upside down and back patted. Healthcare providers looked into airway and baby's airway was cleared with a suction bulb device if secretions and baby's head aligned in neutral position to facilitate airway opening. Head was hyperextended during the resuscitation in four (3.3%) newborns. Importantly, a few (*n* = 6, 4.9%) newborns required prolonged suctioning with a bulb suction device for over 10 minutes.

Less than half (*n* = 30, 45.5%) of the newborns did not initiate spontaneous breathing after the initial BMV for a minute and required reevaluation of the heart rate and help from another provider for improved BMV. Improved BMV entailed repositioning the head and reapplying the mask, clearing secretions and opening the mouth slightly, and squeezing the bag harder with checking the heart rate as per the national resuscitation and HBB protocols. Administration of intravenous 10% dextrose through the newborn's umbilical vein was observed in nine (6.5%) cases who needed ventilation support. The practise of intravenous administration of dextrose was done directly with the use of a 10 cc syringe and needle in the vein without the umbilical vein catheters.

### 3.3. Performance Mean Scores for Each of the Resuscitation Steps

*Preparation for Resuscitation*. Overall performance mean score for preparation for resuscitation was (0.79, SD ± 0.37). The least performed step was identifying a helper before the resuscitation (mean score 0.52, SD ± 0.5).

*Drying/Stimulation*. Nearly all (88%) the newborns were dried gently by rubbing the back with the towel. However, in 31% of babies, the wet towel was not removed. Twenty-nine percent of the newborns were left exposed and not wrapped in dry cloth even at the resuscitation warmer. 

*Airway*. The airway was cleared for all (*n* = 123) newborns who did not respond to stimulation with any form of airway secretions. However, the least performed step (*n* = 23, 40%) was suctioning before stimulation in presence of meconium as per the national guidelines. 

*Bag and Mask Ventilation (BMV)*. Bag and mask ventilation was initiated for all the newborns who did not initiate breathing after airway clearance (*n* = 66, 100%). BMV was initiated within the Golden minute (less than 60 s) in just over half (*n* = 36, 54.6%) of the newborns who required help. The mean time for initiation of bag valve and mask ventilation was 69.2 seconds (SD ± 19.6). Calling for help after initiating bag and mask ventilation was observed in almost all newborns who did not establish breathing after initial BMV (*n* = 26, 87%). Out of those who did not respond to initial BMV, slightly over half (*n* = 18, 60%) of the newborns were ventilated using the bag and mask within the recommended 30–50 breaths per minute. Heart rate response to ventilation was checked by feeling the umbilical cord pulse or in severe cases when cord not pulsating; this was achieved by listening to the heartbeat with a stethoscope. Heart rate evaluation was conducted for almost three-quarters (*n* = 22, 73%) of the newborns who required improved ventilation. In addition, a correct size mask was used on almost all (*n* = 25, 83%) newborns who did not respond to initial BMV.

#### 3.3.1. Cardiopulmonary Resuscitation and/or Advanced Ventilation

Chest compressions with effective breaths were performed for 20 (83.3%) newborns who had poor or no breathing with a low heart rate (less than 60 beats per minute) after improved BMV. One effective breath for every three chest compressions for a minute was administered. Airway, breathing, and heart rate were reassessed every 1-2 minutes during this cardiopulmonary resuscitation. Compressions were stopped after establishment of an improved heart rate (increased pulsation or more than 60 beats per minute) and breathing was supported by providing supplemental oxygen. Twenty-one (87.5%) of the newborns required and were commenced on supplemental oxygen through the nasal catheters after both BMV and chest compressions as per the national guidelines (Table 2).

### 3.4. Quality of Care Scores for the Specific Processes of Neonatal Resuscitation

**Figure 3:**
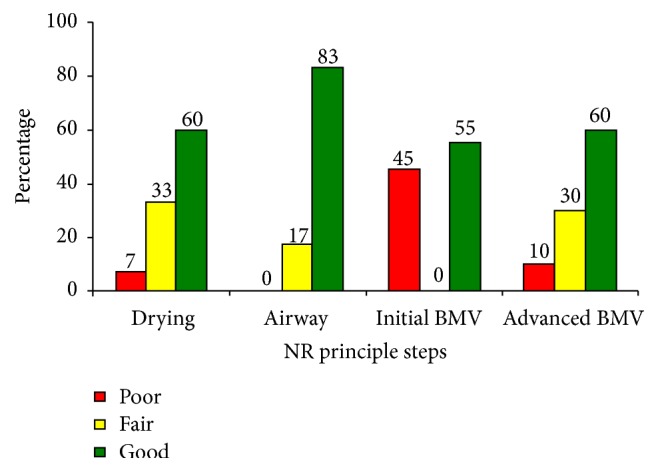
Quality of care scores for the 4 principle steps in neonatal resuscitation. BMV: bag and mask ventilation.

Overall, the QoC scores were high (good) for airway clearance (83%), fair for drying/stimulation & advanced BMV (60%), and poor for the initial BMV commencement within the Golden minute (45%). For those who did not establish breathing after initial BMV, all the recommended five steps were performed in over half (60%) of the newborns (Figure 3).

### 3.5. Association between the HCPs Characteristics and the Quality of Care Scores during NR

Ordered logistic regression illustrated that HCPs with 1–5 years maternity experience providing NR services (drying/stimulation) of good quality was almost twice more than for HCPs with less than a year maternity experience when the other variables in the model were held constant (*P* = 0.003, *β* = 1.860, CI = 0.626–3.093). For airway maintenance, HCPs with 1–5 years of experience working in maternity were twice more likely to provide good quality of care compared to those with less than a year working in maternity (*P* = 0.009, *β* = 1.887, CI = 0.469–3.305) when other factors were held constant. Similarly, those with over 5 years working in maternity were three times more likely to provide good quality of care compared to those with less than a year of working in the unit (*P* = 0.039, *β* = 2.5, CI = 0.127–4.859). For initial BMV, going from a medical officer (doctor) to a nurse/midwife, there was a twofold decrease in the log-odds of the quality of NR care provided, holding all other variables constant (*P* = 0.05, *β* = −2.338, CI = −4.732–0.056) (Table 3).

## 4. Discussion 

### 4.1. Structural Factors

Newborn resuscitation is an essential skill that all HCPs involved in the delivery process must have. The basic essential NR equipment for provision of newborn warmth, airway maintenance, and ventilation was available, accessible, and functional at the resuscitation station. At least two sets of equipment were available in case of multiple births, or for other births occurring at the same time, or in case one set does not function as recommended [34]. In addition, presence of NR guidelines and action plans at the resuscitation stations ensure that standardized NR procedures are performed for all newborns born with birth asphyxia [16, 30]. This finding showed that the hospital is well prepared and ready for NR services although HCPs still miss out on key aspects of resuscitation care: removing the wet towel after drying the newborn, suctioning meconium before stimulation/drying the newborn, and initiation BMV within the Golden minute. This points to the need for proper training of HCPs having the equipment for improved NR performance as recommended by other studies in the country [25, 35].

Years worked in maternity were shown to be associated with good quality scores for drying and airway maintenance and positioning during NR. With longer periods of practice within the high risk referral hospital in the same maternity unit, the HCPs clinically practice and enhance their self-efficacy and competence in NR skills with improved neonatal outcomes [2]. It is expected that those with more years of experience working in the unit help impart the same knowledge and skills through apprenticeship to the new HCPs who join the service along the continuum [36].

Ironically, our findings show no statistically significant association between the HCPs' previous NR training and quality of NR care at the key principle resuscitation steps. Besides, completion of resuscitation training does not imply that an individual is competent to perform NR as demonstrated by the American Heart Association (AHA) neonatal resuscitation programme (NRP) [28]. It has however been demonstrated elsewhere that training courses in NR can effectively increase the competency of HCPs in conducting NR and reducing potentially harmful practices [18, 21, 22]. There is evidence that NR training alone may not be enough to ensure change in practice and retention of skills but may need to be followed up with regular refresher training, as frequently as every 6 months to prevent loss of skills acquired [21, 37–40].

Cleaning and decontamination of NR equipment skills/practices were good among the HCPs. Adequate infection prevention supplies for NR would prevent infections among resuscitated neonates due to their immature immunity [41]. This is important as it helps minimize the neonatal infections/sepsis, the third leading cause of neonatal mortality behind birth asphyxia and prematurity in Kenya [14].

### 4.2. Processes during Neonatal Resuscitation

HCPs performed strongly during the preparation for resuscitation steps to ensure that the newborns at risk receive the emergency attention immediately without delays after delivery. However, identifying a helper before the resuscitation commences was below expectations indicating that there is a weakness among HCPs in recognizing that every baby is at risk of birth asphyxia as recommended [6].

Keeping the newborns warm was a key missing step in early neonatal care among a good number of newborns. It was surprising that some HCPs forget to remove the wet cloth used for drying the newborn. This predisposes the newborns to heat loss through convection leading to hypothermia [42]. Inadequate dry towels in the delivery packs in the ward could explain why some HCPs fail to cover the newborns to keep them warm despite being at the warm resuscitaire after delivery. Evidence has shown that interventions including keeping the baby warm around the time of childbirth have the greatest effect on reducing neonatal mortality, as low coverage and poor quality of health care at that time account for high rates of newborn mortality [6].

Checking the airway for secretions and clearance of obstructive secretions for babies who fail to initiate spontaneous breathing after drying was done for almost all newborns who did not respond after drying as recommended [29]. However, our findings demonstrated that NR skills were poor in airway clearance in presence of meconium, a predictor of birth asphyxia [24, 25, 35]. HCPs dried/stimulated newborns before airway clearance in presence of meconium in nonbreathing newborns as recommended by both the national and the international guidelines [16, 29, 30]. Clearing airway in meconium presence in newborns not breathing should be done before drying the baby thoroughly as meconium inhaled into the lungs can cause breathing problems [30].

Inappropriate positioning of the baby's airway, turning baby upside down, and hyperextending the jaw were observed in a considerable number of newborns. These harmful practices have also been reported in previous studies conducted in the country [24, 25, 35]. This indicates that despite all of the HCPs at least having some form of NR training, there is need for further emphasis on practical NR training to help eradicate some of these potentially harmful practices during NR. A study done in Kenya revealed that despite some HCPs attending NR training, half of them miss out on the practical exposure of the skills [36]. The same author observed that some HCPs had inadequate medical training in NR.

Bag and mask ventilation was initiated for all newborns who did not establish breathing after drying and airway clearance (with secretions). This shows that HCPs clearly recognize the indication for bag and ventilation in newborns without or gasping respirations as recommended [29, 30]. However, there was a delay (more than 60 s) in initiating this key treatment for almost half of the newborns with no breathing after initial interventions. This indicates that HCPs' understanding of the importance of initiating BMV within the Golden minute after birth is poor. Evidence indicates that there is significant improvement in myocardial function and cerebral oxygenation when BMV is initiated within the Golden minute since the period of asphyxia before birth is variable and not precisely known in most cases [29]. When breathing is delayed, the window of opportunity to reverse the consequences of asphyxia (reductions in important blood pressure and cerebral blood flow and cardiac arrest) is small.

This study conspicuously revealed that nurses/midwives (whether diploma or degree holders) who provide the majority of the primary NR were poor (delayed initiating BMV; ventilation rate not within the recommended 30–50 breaths/minute; inconsistent monitoring of the heart rate; BMV provided ineffective as more babies required reevaluation and reapplication of the bag and mask to achieve ventilation) in initial BMV NR skill compared to the doctors (*β* = −2.338, *P* = 0.05, 95% CI = −4.732–0.056). As recommended by WHO and HBB, monitoring the heart rate is the most important determinant for assessing the response to resuscitative interventions [29, 30]. Failure by nurses to consistently monitor the heart rate and provide BMV within the recommended rate indicates either lack of formal practical training in neonatal care or need for skills improvement with repetitive training with a skilled person. Regarding the fact that nursing education takes almost half the duration of time it takes to train doctors may explain constrained time for training [36]. This points to the urgent need for regular refresher training for nurses/midwives within 12 months of training and practice.

Most HCPs called for help from other colleagues in the unit in cases of failed initial BMV. This is an important step in the collaborative management in a hospital. Calling for help from more experienced colleagues and other cadres trained in NR helps increases the likelihood of survival of the neonate. This help could include assistance with BMV, airway maintenance, and checking of heart rate that determines the continuation or stoppage of the NR [16, 29].

This study further demonstrated an existing gap in knowledge and skill on the rate of bag and mask ventilation and the implication of observable chest rise with each ventilation as recommended [16, 29, 30]. This finding has also been reported in other studies done in Kenya [24, 25, 43]. Squeezing the bag gently within the 30–50 breaths per minute to produce gentle movement of the chest as if the baby was taking an easy breath ensures that there is no air leak between the mask and the baby's face [30]. This implies that HCPs do not clearly understand the physiology of the normal newborn respiration pattern and, as such, many newborns may suffer further damage due to inappropriate BMV.

Encouragingly, many newborns with poor or gasping breathing after unsatisfactory BMV were provided with chest compressions and/or supplemental oxygen. This indicates that HCPs understand the importance of the priority of giving adequate ventilation by bag and mask before commencing on chest compressions and/or supplemental oxygen. Importantly, if ventilation is performed correctly, chest compressions are rarely indicated (1 in 1000) [33].

Therefore, clearly to improve the quality of care provided during NR, efforts must address both the healthcare providers and the health facilities.

### 4.3. Strengths of the Study

Conducting direct observations allowed us to see what HCPs exactly do rather than relying on what they say they did. Secondly, conducting multiple observations (3–5) allowed us to eliminate possible anxiety that could be associated with direct observations on the part of the HCP being observed and give a true representation of the HCP. Using research assistants from the same maternity unit helped us to minimize the expected Hawthorn' effect with direct observations.

### 4.4. Study Limitations

There was a risk of the observer to feel compelled to assist during the resuscitation and bias since the research assistants were recruited from the antenatal ward of the hospital. This was minimized by training of the RAs to understand the study procedures and supervision. The HCPs knew why the researchers were there. As a result, a change in behaviour due to the researchers' presence was anticipated. The assumption was that, after a few minutes, the HCP would become accustomed to the researchers presence and function in a more natural fashion, but further capturing the events more than once in a resuscitation offered an opportunity to minimize this risk. This is a major drawback of the observation studies; however, this helped to observe the “where” and “when” of the ongoing process/situation/behaviour wanted not relying on reported information. Inconsistent evaluation of the heart rate by use of stethoscope for monitoring response to resuscitative interventions by the HCP was negated by observing the immediate outcomes of resuscitation, for example, change in skin colour, active withdrawal/crying, and active motion by the newborn. Few HCPs involved in resuscitation were observed a multiple times to achieve the sample size of the resuscitation observations. Therefore, our results should be interpreted in light of the small sample size.

### 4.5. Implications of the Findings

This study has implications both at the health facility level and the government level for the fight against neonatal mortality due to birth asphyxia. A few newborns miss out on the most important step: being kept warm adequately and this could result in further neonatal deaths precipitated by hypothermia. Secondly, many newborns with birth asphyxia are denied the vital ventilation within the Golden minute and this could be a signal for other sequelae associated with birth asphyxia. Caring for newborns with meconium in airway, a predictor of birth asphyxia is still poor. Health facilities should invest more in ensuring that the initial steps of drying, warmth, airway maintenance, and BMV within the Golden minute are perfectly performed to prevent more newborns proceeding to later stages of resuscitation that are associated with less survival.

### 4.6. Conclusion and Recommendation

The hospital is prepared in both healthcare providers and equipment to provide NR. However, training in neonatal resuscitation for HCPs is poorly spaced allowing deterioration of key skills in maintaining the warm chain, airway maintenance, ventilation, and circulation. Conspicuously, inappropriate practices are still performed by all the HCPs irrespective of cadre. Healthcare providers need cost-effective initial and regular refresher NR training and clinical mentorship with a focus on skills retention at least every year.

## Figures and Tables

**Table 1:** Background characteristics of HCPs with the distribution of newborns resuscitated at KCGH.

HCP characteristics	Frequency (*n* = 28)	Percentage (%)	Newborns resuscitated (*n* = 138)	Percentage (%)
*Age (years)*				
<25 years	3	10.7	15	10.9
>25–50 years	25	89.3	123	89.1
*Sex *				
Male	8	28.6	38	27.5
Female	20	71.4	100	72.5
*Professional cadre *				
Nurses/midwives	20	71.4	100	72.5
Medical officers	4	14.3	20	14.5
Anaesthetists	3	10.7	13	9.4
Clinical officers	1	3.6	5	3.6
*Qualification *				
Diploma	15	53.6	73	52.9
Bachelor degree	13	46.4	65	47.1
*Previous training in NR*				
Yes	18	64.3	88	63.8
No	10	35.7	50	36.2
*Period since the last NR training (n = 18)* ^*∗*^			*(n = 88)*	
<6 months	6	33.3	30	34.1
≥Over 12 months	12	66.7	58	65.9
*Support supervision in NR*				
Yes	25	89.3	123	89.1
No	3	10.7	15	10.9
*Period since last support supervision in NR (n = 25)*			*(n = 123)*	
Past 6 months	9	36.0	45	32.6
>6–12 months	6	24.0	30	21.7
>12 months	10	40.0	48	34.8
*Period working in maternity*				
<1 year	3	10.7	15	10.9
>1 year–5 years	17	60.7	83	60.1
≥5 years	8	28.6	40	29.0

^**∗**^No HCP trained within last 6–12 months.

**Table 2:** Performance mean scores for each of the neonatal resuscitation steps.

Step in NR	Observations	Mean score (0-1)	SD (±)
Preparation for resuscitation			
Preparation for resuscitation area	138	0.88	0.32
Check NR equipment availability	138	0.88	0.33
Check NR equipment functioning	138	0.88	0.33
Identify a helper	138	0.52	0.5
*Overall mean score*		*0.79*	
Drying/stimulation^*∗*^			
Baby dried thoroughly by gently rubbing the back	138	0.88	0.32
Wet cloth removed	138	0.69	0.46
Baby kept warm	138	0.71	0.46
*Overall mean score*		*0.76*	
Airway clearance^*∗∗*^			
Looked into airway	123	0.98	0.13
If meconium, suctioning done before stimulation	57	0.4	0.49
Airway cleared with suction bulb if unresponsive	123	1	0
Baby's head in neutral position	123	0.83	0.38
*Overall mean score*		*0.80*	
Bag and mask ventilation for breathing^#^			
** **Initial			
** **BMV initiated	66	1.00	0.00
** **BMV initiated within the Golden minute	66	0.55	0.5
** **Advanced			
HCP call for help	30	0.87	0.35
Correct mask size used during BMV	30	0.83	0.37
Chest movements observed with each ventilation	30	0.63	0.49
BMV rate within 30–50 breaths/minute	30	0.60	0.50
Baby's HR checked at 1 min	30	0.73	0.45
*Overall mean score*		*0.74*	
Advanced ventilation^##^			
Effective breath with chest compressions	24	0.83	0.38
Supportive oxygen	24	0.88	0.34
*Overall mean*		*0.86*	

^*∗*^15 babies responded to drying, ^*∗∗*^57 babies responded to airway clearance, ^#^36 babies responded to BMV, and ^##^11 responded to supportive oxygen and/or chest compressions. BMV: bag and mask ventilation; HCP: healthcare provider; HR: heart rate.

**Table 3:** Ordered logistic regression of the QoC scores with HCPs' characteristics.

HCP characteristics	*β*	*P* value	95% CI
Drying/stimulation			
Professional cadre			
Doctors (ref)			
Nurses	0.81	0.203	−0.438–2.065
C/Officer	−0.96	0.521	−3.904–1.977
Anesthetists	1.51	0.127	−0.429–3.451
NR training			
No (ref)			
Yes	0.325	0.555	−0.755–1.404
Maternity experience			
<1 year (ref)			
1–5 years	1.860	0.003^**∗**^	0.626–3.093
>5 years	1.566	**0.063**	−0.087–3.219
Support supervision			
No (ref)			
Yes	−1.769	0.123	−4.018–0.480

Airway maintenance			
Professional cadre			
Doctors (ref)			
Nurses	−0.693	0.568	−3.071–1.685
C/Officer	0.347	1.000	−18953–18954
Anesthetists	16.135	0.997	−9029–9061
NR training			
No (ref)			
Yes	0.492	0.525	−1.027–2.012
Maternity experience			
<1 year (ref)			
1–5 years	1.887	0.009^**∗**^	0.469–3.305
>5 years	2.493	0.039^**∗**^	0.127–4.859
Support supervision			
No (ref)			
Yes	−16.199	0.997	−10014–9982

Initial BMV			
Professional cadre			
Doctors (ref)			
Nurses	−2.338	0.05^**∗**^	−4.732–0.056
C/Officer	−19.396	0.990	−3173–3134
Anesthetists	−2.811	0.100	−6.163–0.541
NR training			
No (ref)			
Yes	−0.258	0.742	−1.797–1.280
Maternity experience			
<1 year (ref)			
1–5 years	0.341	0.681	−1.283–1.964
>5 years	0.221	0.852	−2.105–2.546
Support supervision			
No (ref)			
Yes	−16.22	0.992	−3170–3137

Advanced BMV			
Professional cadre			
Doctors (ref)			
Nurses	−15.658	0.997	−7047–7016
C/Officer	−2.400	1.000	−14917–14917
NR training			
No (ref)			
Yes	1.956	0.148	−0.694–4.606
Maternity experience			
<1 year (ref)			
1–5 years	0.992	0.434	−1.492–3.477
>5 years	−1.584	0.377	−5.097–1.928
Support supervision			
No (ref)			
Yes	−2.576	1.000	−11121–11116

^*∗*^ *P* ≤ 0.05 statistically significant.
